# Taxane-based chemotherapy and risk of breast cancer-related lymphedema: Protocol for a systematic review and meta-analysis

**DOI:** 10.1097/MD.0000000000016563

**Published:** 2019-07-26

**Authors:** Zhenhua Zhang, Xiwen Zhang, Shuntai Chen, Juling Jiang, Runzhi Qi, Xing Zhang, Yupeng Xi, Meng Li, Honggang Zheng, Baojin Hua

**Affiliations:** a Beijing University of Chinese Medicine; b Department of Oncology, Guang’anmen Hospital, China Academy of Chinese Medical Sciences, Beijing, China.

**Keywords:** breast cancer-related lymphedema, protocol, systematic review, Taxane-based chemotherapy

## Abstract

**Background:**

Many studies were performed to explore the correlation between taxane-based chemotherapy and the risk of breast cancer-related lymphedema (BCRL), however, with inconsistent results. Hence, the purpose of this study is to evaluate whether taxane-based chemotherapy is a risk factor for BCRL.

**Methods:**

A comprehensive systematic search of clinical trials published in the PubMed, Embase and the Cochrane Library databases will be conducted to identify eligible studies up to the date of December 31, 2018. We will employ risk ratios with 95% confidence intervals (95% CIs) to estimate the correlations between taxane-based chemotherapy and BCRL. Meta-analysis will be performed using Stata SE version 12.0 software.

**Results:**

The results of this systematic review and meta-analysis will provide a high-quality synthesis of existing evidence of the correlations between taxane-based chemotherapy and the risk of BCRL.

**Conclusion:**

The protocol will provide updated evidence for the use of taxane-based chemotherapy in postoperative breast cancer patients.

**Ethics and dissemination:**

It is not necessary for ethical approval because it is based on published studies. The protocol will be disseminated in a peer-reviewed journal or presented at a topic-related conference.

**Trial registration:**

This systematic review protocol has been registered with a number of CRD42019123989.

## 1 Introduction

The Global Cancer Statistics showed that breast cancer is the highest cancer incidence and the leading cause of cancer death among women worldwide in 2012, and the breast cancer alone accounts for a quarter of all cancer cases and 15% of cancer deaths among females.^[1]^ In recent decades, with targeted therapy, radiotherapy, chemotherapy, and endocrine therapy developing, the survival of breast cancer patients has been significantly improved. And the clinical focus has been shifted to the alleviation of disease burden and the treatment of chronic complications.^[2]^ One of the most common complications of breast cancer treatment is breast cancer-related lymphedema (BCRL), which is caused by accumulation of lymphatic fluid owing to remodeling of the tissue structure and fibrosis, leading to chronic swelling of the arms, breasts, or torso.^[3]^ And these symptoms may compromise the patient's wellgoing physical condition and decrease the quality of life.^[4,5]^ The well-established risk factors for developing lymphedema are ALND,^[2,6–14]^ regional lymph node radiation,^[6,8,13,15]^ and higher body mass index.^[7,8,14]^The association between chemotherapy agents and lymphedema has been discussed in many studies,^[5]^ but the results are inconsistent. Taxane-based chemotherapy is the conventional treatment for breast cancer and can significantly improve progression-free survival and overall survival of patients.^[16,17]^ Taxane-based chemotherapy, especially docetaxel, results in fluid retention in extremities because of the increase of the extracellular fluid.^[18,19]^ Although many published studies have discussed the correlation between taxane-based chemotherapy and the risk of BCRL, owing to differences in research methods, sample size, and study population, the results of a single study are difficult to generalize to the entire population, resulting in inconsistent conclusions. Swaroop et al^[20]^ believed that taxane-based chemotherapy did not increase the risk of lymphedema in patients, whereas Cariati et al^[21]^ concluded that paclitaxel adjuvant chemotherapy played a key role in the generation of BCRL in postoperative patients. The purpose of this study is to evaluate whether taxane-based chemotherapy is a risk factor for BCRL through a comprehensive and systematic meta-analysis.

## 2 Methods

### 2.1 Study registration

The protocol for this systematic review and meta-analysis has been registered in the International Prospective Register of Systematic Reviews (PROSPERO) platform with the registration number of CRD42019123989. And this protocol was performed in accordance with the Preferred Reporting Items for Systematic Reviews and Meta-Analyses Protocols (PRISMA-P) statement guidelines and the Systematic Reviews and Meta-Analyses will be reported based on the PRISMA guidelines. Ethical approval is unnecessary because this is a literature-based study.

### 2.2 Data sources and search strategy

The search strategy for this study is based on Meta-Analysis of Observational Studies in Epidemiology (MOOSE) and PRISMA statement. The 2 authors (SC and JJ) will independently conduct a comprehensive systematic search of clinical trials published on electronic databases such as the PubMed, Embase, the Cochrane Library databases and the ClinicalTrails.gov up to the date of December 31, 2018. The following terms will be used: “breast neoplasms OR breast cancer OR breastcancer OR breast tumor OR breast tumour OR mammary neoplasm OR mammary carcinoma OR breast malignan OR breast metastas OR mammary malignan OR mammary metastas OR breast neoplasm OR breast carcinoma” and “lymphoedema OR lymphedema OR lymphedema OR lymphatic edema OR oedema OR edema OR edema OR swelling OR elephantias” and “diterpenes OR paclitaxel OR docetaxel OR cabazital OR toxoids.” The detailed retrieval strategy for Pubmed is illustrated in Table 1. This search strategy contains all the search terms, and the other strategies of Embase and the ClinicalTrails.gov will be conducted based on these results. As there is currently no RCT for this type of study, only observational studies were included. In addition, we will examine reference lists of all articles that meet our requirements to prevent omissions.

**Table 1:**
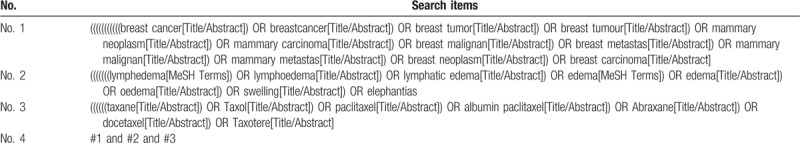
Search strategy used in PubMed.

### 2.3 Inclusion and exclusion criteria

#### 2.3.1 Types of studies

Published observational cohort and case–control studies will be included without limiting the sample size and type of breast cancer. To avoid duplication, identical or overlapping studies will be included as one study with the sample size as the inclusion criteria. Case reports, case series, letters to the editor, conference abstracts, reviews, meta-analyses, and animal experiments will be excluded.

#### 2.3.2 Types of participants

Women eligible for inclusion were clinically diagnosed with unilateral breast cancer based on histopathology and had undergone therapeutic breast surgery. But there are no restrictions between pathological stages, ages, countries, ethnic, economy, education backgrounds.

#### 2.3.3 Types of interventions

Taxane-based chemotherapy includes paclitaxel (Taxol), albumin paclitaxel (Abraxane), and docetaxel (Taxotere). These 3 reagents are suitable for use alone or in combination with other chemotherapy drugs.

#### 2.3.4 Types of Comparison

Patients in the control group are those who have not received taxane-based chemotherapy after unilateral breast cancer surgery with or without chemotherapy with other reagents.

#### 2.3.5 Types of outcomes

The primary outcome was the incidence of BCRL. Owing to the lack of clear diagnosis and evaluation criteria for lymphedema, this study accepted objective measurement of arm circumference and subjective feelings (such as questionnaire) as the diagnostic criteria. The follow-up time should not be <6 months, and the number and interval of follow-up time should not be limited. The secondary outcomes will be the incidence of BCRL in different taxane-based chemotherapy interventions, study designs. Results of lymphedema will use a binary classification (presence or absence).

### 2.4 Selection of studies

**Figure 1:**
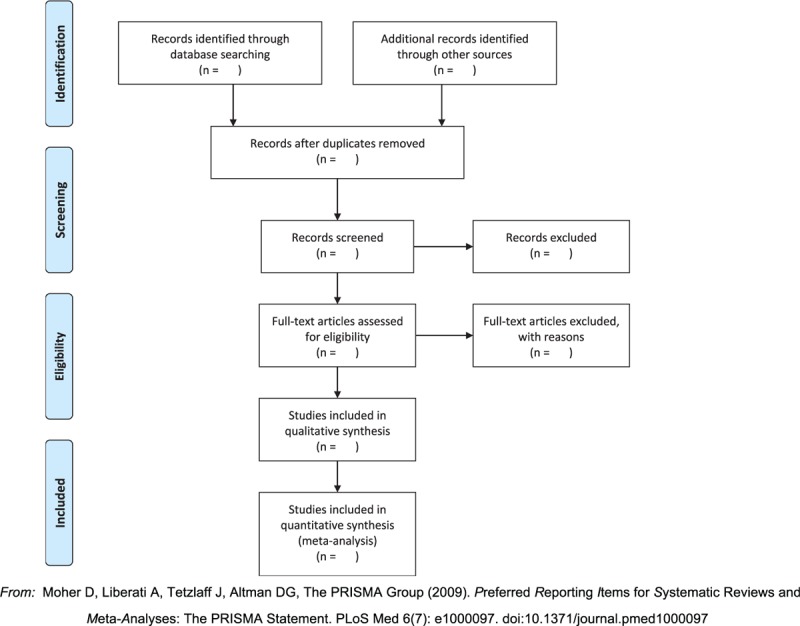
Flow of information through the different phases of a systematic review. From: Moher D, Liberati A, Tetzlaff J, Altman DG, The PRISMA Group (2009). Preferred Reporting Items for Systematic Reviews and Meta-Analyses: The PRISMA Statement. PLoS Med 6(7): e1000097. doi:10.1371/journal.pmed1000097. For more information, visit www.prisma-statement.org.

All review authors will receive training to understand the purpose and requirements of this review before selecting studies. Two review authors (RQ and XZ) will independently complete the retrieval of the titles, abstract, and keywords of all possible eligible articles. The eligible full text of the study was then downloaded and further evaluated independently by the 2 reviewers based on inclusion and exclusion criteria. Disagreements between the 2 review authors will be resolved by discussion, or a third author (ZZ) will be sought. The PRISMA flow chart (http://www.prisma-statement.org) will be used to show the literature retrieval process of this study, as shown in Figure 1.

### 2.5 Data extraction and management

The 2 review authors (RQ and XZ) will complete the standard data extraction form independently to complete data extraction of the included studies. The information extracted included: author, year of publication, type of study, sample size, definition of lymphedema, chemotherapy drugs, and follow-up time. We will contact the corresponding author, if the content to be extracted cannot be obtained through the full text. Any disagreements will be settled through discussion or an arbiter (ZZ).

### 2.6 Assessment of quality in included studies

The quality assessment of the included study will be performed independently by 2 review authors (XZ and ML) using the Newcastle–Ottawa Quality Assessment Scale (NOS), which contains 3 dimensions including selection, comparability, and—depending on the study type—outcome (cohort studies) or exposure (case–control studies).^[22]^ The total score of NOS in the study is ≥5 points, which is considered as a medium-high quality study; otherwise, it is a low-quality study. Any dispute will be settled through discussion or a third arbiter (ZZ).

### 2.7 Data synthesis

Analysis will be performed using Stata SE version 12.0. The pooled risk ratio with the corresponding 95% confidence intervals for all studies will be computed. If there is heterogeneity between studies, the random-effect model will be used for calculation. Otherwise, the fixed-effect model will be adopted. If heterogeneity exists, we will conduct sensitivity and subgroup analysis to explore the reasons for its existence.^[23]^ If these data cannot be combined into a meta-analysis, we will summarize the description of this study.

### 2.8 Assessment of heterogeneity

Cochran *Q* test and Higgins *I*^2^ method will be used to evaluate the heterogeneity. If *P* value is <.1 or *I*^2^ value is >50%, we believe there is heterogeneity between studies.

### 2.9 Assessment of publication biases

Begg funnel plot and Egger test will be employed to assess the publication bias. Begg funnel plot is drawn using the logarithm of odds ratio (OR) (log OR) as the abscissa and the inverse of the standard error of OR (1/SE[log OR]) as the ordinate. The Egger test is a test for linear regression to measure the symmetry of the funnel chart according to log OR. An asymmetric funnel plot or a *P* value <.1 indicates the publication bias.

### 2.10 Subgroup analysis

Subgroup meta-analysis will be used to explore possible factors that may cause high heterogeneity. In cases of high heterogeneity, we will conduct subgroup analysis according to the following aspects: the differences between the study design and interventions (paclitaxel, albumin paclitaxel, and docetaxel).

### 2.11 Sensitivity analysis

We will conduct sensitivity analysis with sequentially excluding each study and combining the remaining studies to find the impact of each study on the overall results.

## 3 Discussion

Taxane-based chemotherapy is a routine treatment for breast cancer patients after surgery. BCRL is also a common complication that affects the quality of life of patients. A meta-analysis^[7]^ shows that the postoperative incidence is 20%. In this study, a systematic and comprehensive analysis will be conducted to determine whether taxane-based chemotherapy is a risk factor for postoperative lymphedema in breast cancer patients and provide evidence for clinical chemotherapy. However, there are some limitations that need to be pointed out. First, the inconsistency of BCRL measurements may increase the heterogeneity of the study. Second, only studies published in English will be included, which may increase publication bias.

## Author contributions

**Data curation:** Shuntai Chen, Juling Jiang, Runzhi Qi, Xing Zhang, Yupeng Xi, Meng Li.

**Supervision:** Honggang Zheng, Baojin Hua.

**Writing – original draft:** Zhenhua Zhang, Xiwen Zhang.

**Writing – review & editing:** Zhenhua Zhang.

## References

[1] Torre LA Bray F Siegel RL Global cancer statistics, 2012. CA Cancer J Clin 2015;65:87–108. 25651787 10.3322/caac.21262

[2] Asdourian MS Skolny MN Brunelle C Precautions for breast cancer-related lymphoedema: risk from air travel, ipsilateral arm blood pressure measurements, skin puncture, extreme temperatures, and cellulitis. Lancet Oncol 2016;17:e392–405. 27599144 10.1016/S1470-2045(16)30204-2

[3] Maclellan RA Greene AK Lymphedema. Semin Pediatr Surg 2014;23:191–7. 25241097 10.1053/j.sempedsurg.2014.07.004

[4] Ahmed RL Prizment A Lazovich D Lymphedema and quality of life in breast cancer survivors: the Iowa Women's Health Study. J Clin Oncol 2008;26:5689–96. 19001331 10.1200/JCO.2008.16.4731 PMC2600601

[5] Lee SH Min Y-S Park HY Health-related quality of life in breast cancer patients with lymphedema who survived more than one year after surgery. J Breast Cancer 2012;15:449–53. 23346175 10.4048/jbc.2012.15.4.449 PMC3542854

[6] Tsai RJ Dennis LK Lynch CF The risk of developing arm lymphedema among breast cancer survivors: a meta-analysis of treatment factors. Ann Surg Oncol 2009;16:1959–72. 19365624 10.1245/s10434-009-0452-2

[7] DiSipio T Rye S Newman B Incidence of unilateral arm lymphoedema after breast cancer: a systematic review and meta-analysis. Lancet Oncol 2013;14:500–15. 23540561 10.1016/S1470-2045(13)70076-7

[8] Ferguson CM Swaroop MN Horick N Impact of Ipsilateral Blood Draws, Injections, Blood Pressure Measurements, and Air Travel on the Risk of Lymphedema for Patients Treated for Breast Cancer. J Clin Oncol 2016;34:691–8. 26644530 10.1200/JCO.2015.61.5948 PMC4872021

[9] Sayegh HE Asdourian MS Swaroop MN Diagnostic methods, risk factors, prevention, and management of breast cancer-related lymphedema: past, present, and future directions. Curr Breast Cancer Rep 2017;9:111–21. 28894513 10.1007/s12609-017-0237-8 PMC5590641

[10] McLaughlin SA Wright MJ Morris KT Prevalence of lymphedema in women with breast cancer 5 years after sentinel lymph node biopsy or axillary dissection: objective measurements. J Clin Oncol 2008;26:5213–9. 18838709 10.1200/JCO.2008.16.3725 PMC2652091

[11] Kilbreath SL Refshauge KM Beith JM Risk factors for lymphoedema in women with breast cancer: A large prospective cohort. Breast 2016;28:29–36. 27183497 10.1016/j.breast.2016.04.011

[12] Gartner R Jensen MB Kronborg L Self-reported arm-lymphedema and functional impairment after breast cancer treatment–a nationwide study of prevalence and associated factors. Breast 2010;19:506–15. 20561790 10.1016/j.breast.2010.05.015

[13] Warren LE Miller CL Horick N The impact of radiation therapy on the risk of lymphedema after treatment for breast cancer: a prospective cohort study. Int J Radiat Oncol Biol Phys 2014;88:565–71. 24411624 10.1016/j.ijrobp.2013.11.232 PMC3928974

[14] Meeske KA Sullivan-Halley J Smith AW Risk factors for arm lymphedema following breast cancer diagnosis in Black women and White women. Breast Cancer Res Treat 2009;113:383–91. 18297429 10.1007/s10549-008-9940-5

[15] Haddad P Farzin M Amouzegar-Hashemi F A multicentre cross-sectional study of arm lymphedema four or more years after breast cancer treatment in Iranian patients. Breast Cancer 2010;17:281–5. 19789952 10.1007/s12282-009-0165-1

[16] Henderson IC Berry DA Demetri GD Improved outcomes from adding sequential Paclitaxel but not from escalating Doxorubicin dose in an adjuvant chemotherapy regimen for patients with node-positive primary breast cancer. J Clin Oncol 2003;21:976–83. 12637460 10.1200/JCO.2003.02.063

[17] Martín M Seguí MA Antón A Adjuvant docetaxel for high-risk, nodenegative breast cancer. N Engl J Med 2010;363:2200–10. 21121833 10.1056/NEJMoa0910320

[18] Brønstad A Berg A K RR Effects of the taxanes paclitaxel and docetaxel on edema formation and interstitial fluid pressure. Am J Physiol Heart Circ Physiol 2004;287:H963–8. 15059777 10.1152/ajpheart.01052.2003

[19] Semb KA Aamdal S Capillary protein leak syndrome appears to explain fluid retention in cancer patients who receive docetaxel treatment. J Clin Oncol 1998;16:3426–32. 9779722 10.1200/JCO.1998.16.10.3426

[20] Swaroop MN Ferguson CM Horick NK Impact of adjuvant taxane-based chemotherapy on development of breast cancer-related lymphedema: results from a large prospective cohort. Breast Cancer Res Treat 2015;151:393–403. 25940996 10.1007/s10549-015-3408-1 PMC4432026

[21] Cariati M Bains SK Grootendorst MR Adjuvant taxanes and the development of breast cancer-related arm lymphoedema. Br J Surg 2015;102:1071–8. 26040263 10.1002/bjs.9846

[22] Wells GA Shea B O’Connell D The Newcastle–Ottawa Scale (NOS) for assessing the quality of nonrandomised studies in meta-analyses. Available at: http://www.ohri.ca/programs/clinical_epidemiology/oxford.asp Accessed November 10, 2017.

[23] Higgins JP Thompson SG Deeks JJ Measuring inconsistency in meta-analyses. BMJ 2003;327:557–60. 12958120 10.1136/bmj.327.7414.557 PMC192859

